# Identification of Calculous Pyonephrosis by CT-Based Radiomics and Deep Learning

**DOI:** 10.3390/bioengineering11070662

**Published:** 2024-06-28

**Authors:** Guanjie Yuan, Lingli Cai, Weinuo Qu, Ziling Zhou, Ping Liang, Jun Chen, Chuou Xu, Jiaqiao Zhang, Shaogang Wang, Qian Chu, Zhen Li

**Affiliations:** 1 Department of Radiology, Tongji Hospital, Tongji Medical College, Huazhong University of Science and Technology, Wuhan 430030, China; ygjforever98@163.com (G.Y.); cailinglitjh@163.com (L.C.); quweinuo@hust.edu.cn (W.Q.); zilingzhou@tjh.tjmu.edu.cn (Z.Z.); pinglianglp@163.com (P.L.); zhenli@hust.edu.cn (Z.L.); 2 Bayer Healthcare, Wuhan 430000, China; chencharel@gmail.com; 3 Department of Urology, Tongji Hospital, Tongji Medical College, Huazhong University of Science and Technology, Wuhan 430030, China; sgwangtjm@163.com; 4 Department of Oncology, Tongji Hospital, Tongji Medical College, Huazhong University of Science and Technology, Wuhan 430030, China; qianchu@tjh.tjmu.edu.cn

**Keywords:** pyonephrosis, hydronephrosis, computed tomography, radiomics, convolutional neural network

## Abstract

Urgent detection of calculous pyonephrosis is crucial for surgical planning and preventing severe outcomes. This study aims to evaluate the performance of computed tomography (CT)-based radiomics and a three-dimensional convolutional neural network (3D-CNN) model, integrated with independent clinical factors, to identify patients with calculous pyonephrosis. We recruited 182 patients receiving either percutaneous nephrostomy tube placement or percutaneous nephrolithotomy for calculous hydronephrosis or pyonephrosis. The regions of interest were manually delineated on plain CT images and the CT attenuation value (HU) was measured. Radiomics analysis was performed using least absolute shrinkage and selection operator (LASSO). A 3D-CNN model was also developed. The better-performing machine-learning model was combined with independent clinical factors to build a comprehensive clinical machine-learning model. The performance of these models was assessed using receiver operating characteristic analysis and decision curve analysis. Fever, blood neutrophils, and urine leukocytes were independent risk factors for pyonephrosis. The radiomics model showed higher area under the curve (AUC) than the 3D-CNN model and HU (0.876 vs. 0.599, 0.578; *p* = 0.003, 0.002) in the testing cohort. The clinical machine-learning model surpassed the clinical model in both the training (0.975 vs. 0.904, *p* = 0.019) and testing (0.967 vs. 0.889, *p* = 0.045) cohorts.

## 1. Introduction

Pyonephrosis is regarded as a urological emergency characterized by gross accumulation of pus within the renal collecting system and suppurative destruction of renal parenchyma, which may rapidly develop into renal failure, urosepsis, and uroseptic shock [1,2]. This condition differs from calculous hydronephrosis in that it typically necessitates immediate relief of obstruction, either through nephrostomy or ureteral stent placement, before proceeding with the appropriate surgical technique for stone removal [3,4]. In addition, for cases of occult pyelonephritis without obvious preoperative symptoms, if discovered during surgery, the procedure should be terminated immediately, or as soon as possible, to prevent serious postoperative infection complications. Thus, early or preoperative identification of hydronephrosis and pyonephrosis has critical clinical value, including devising a surgical strategy and preventing life-threatening outcomes.

At present, the identification of pyonephrosis primarily relies on clinical symptoms and urinary assessments for pathogens and white blood cells [5]. However, a considerable number of patients with pyonephrosis present a lack of typical symptoms like high fever, chills, and flank pain due to the abuse of broad-spectrum antibiotics, a decrease in immunity, or recurrent chronic attack, which makes the diagnosis of pyonephrosis a tricky problem [6,7]. Furthermore, a study has found that urine leukocyte counts in some patients with pyonephrosis were only slightly increased or even normal and more than 50% of patients exhibited negative in the examination of urine culture [8], which denotes that urinary evaluation sometimes could not effectively reflect the status of intrarenal infection and accurately differentiate pyonephrosis from uninfected hydronephrosis.

Ultrasound is easily influenced by the patient’s intestinal gas and the physician’s subjective experience, while magnetic resonance imaging has a low sensitivity for detecting stones, longer scanning times, and higher costs. Thus, non-contrast computed tomography (CT) is considered the preferred method for assessing hydronephrosis with the capability to display the degree and underlying cause [9,10,11]. Previous studies have also demonstrated that CT attenuation values (Hounsfield Unit—HU) of the fluid in the dilated renal collecting system may be an effective tool for differentiating calculous pyonephrosis from uninfected hydronephrosis [12,13,14]. However, with the development of precision medicine, more and more studies focus on the deep-level quantitative features in medical images rather than simple grayscale differences [15,16]. Radiomics is an emerging image analysis method that can automatically extract high-throughput quantitative statistical features from conventional medical images to establish a deep connection between radiological data and biological information [17,18]. Machine learning is pivotal in a range of biomedical engineering applications, such as multi-omics, biological and medical imaging, brain and body machine interfaces, material development, fault detection, and system integration etc. [19,20,21,22]. Particularly in the medical field, various machine-learning models are employed for clinical diagnostics, precision treatments, and health monitoring [23]. Among these, convolutional neural networks (CNNs) have attracted substantial interest for their ability to perform lesion detection, localization, segmentation, and classification in medical image analysis [23,24,25]. The three-dimensional convolutional neural network (3D-CNN) can perform image explanation by using a convolutional kernel to recognize both spectral and spatial features and then classify images according to the labels given on the training data [26]. Currently, these methods have been widely used to detect urinary infection stones, analyze the urinary stone composition, and predict the stone-free rate of flexible ureteroscopy [27,28,29], whereas the capability of radiomics and three-dimensional convolutional neural network (3D-CNN) analysis in differentiating calculous pyonephrosis from uninfected hydronephrosis is yet to be explored.

Thus, this study aims to compare the performance of the radiomics model, 3D-CNN model, and HU in identifying patients with calculous pyonephrosis and integrate the best performing one with independent clinical factors to construct a combined model, exploring whether it has added value compared to the clinical model itself. Firstly, we retrospectively enrolled patients and divided them into training and testing cohorts. Then, the lesion segmentation and HU measurement, radiomics analysis (including feature extraction, feature selection, and model construction), and 3D-CNN model training were performed. Lastly, the performance of different models was assessed and the interpretability and practicality of the final model were described.

## 2. Materials and Methods

### 2.1. Patient Selection

**Figure 1:**
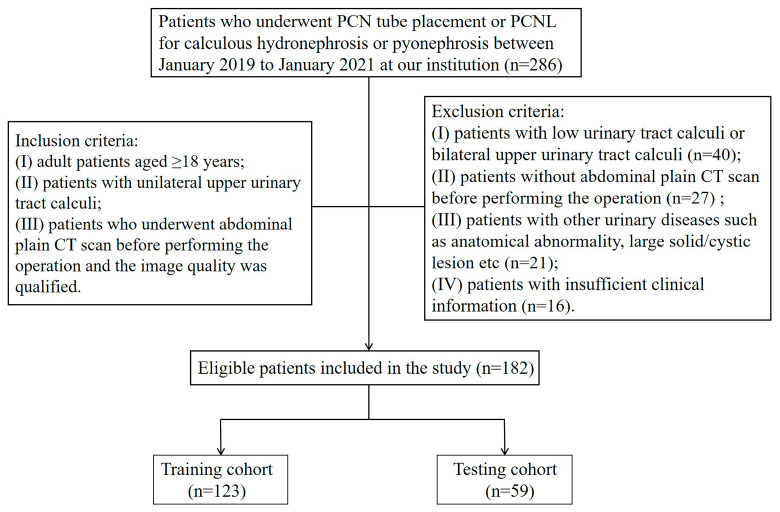
The flowchart for patient selection. PCN, percutaneous nephrostomy; PCNL, percutaneous nephrolithotomy; CT, computed tomography.

This retrospective study was approved by the institutional review board of our hospital and exempted from requiring patient informed consent. Patients receiving percutaneous nephrostomy tube placement or percutaneous nephrolithotomy for calculous hydronephrosis from our institution were selected by searching the medical records for the period January 2019 to January 2021. The inclusion criteria were as follows: (I) adult patients aged ≥18 years; (II) patients with unilateral upper urinary tract calculi; (III) patients who underwent non-contrast abdominal CT scan before performing the operation and the image quality was qualified. The exclusion criteria were as follows: (I) patients with low urinary tract calculi or bilateral upper urinary tract calculi; (II) patients without non-contrast abdominal CT scan before performing the operation; (III) patients with other urinary diseases such as anatomical abnormality, large solid/cystic lesion, etc.; (IV) patients with insufficient clinical information. Finally, a total of 182 patients (84 females/98 males, mean age 53 ± 13 years; age range, 23–86 years) were enrolled. These participants were randomly divided into two independent cohorts: training cohort (*n* = 123) and testing cohort (*n* = 59), based on a 7:3 ratio. The flowchart of patient selection is listed in Figure 1.

### 2.2. Confirmation of Pyonephrosis, Clinical Data Collection, and Clinical Model Building

The percutaneous nephrostomy or percutaneous nephrolithotomy procedure was performed by experienced urologists at our hospital, with pyonephrosis confirmed by the presence of pyuria following the needle insertion [30]. Preoperative clinical characteristics, including basic demographic data (age and gender), body mass index, clinical symptoms (fever and renal colic), coexisting conditions (hypertension and diabetes), history of stone surgery, stone characteristics (laterality, location, and size), hydronephrosis or pyonephrosis levels, the laboratory variables of blood and urine, were obtained by reviewing medical records. The degree of hydronephrosis or pyonephrosis was classified as mild, moderate, and severe according to Noble’s grading system [31]. Urine culture with a single microorganism growth of 10^5^ colony forming units/mL for a sterile midstream urine sample and 10^4^ colony forming units/mL for a catheterized sample were considered positive results [32].

Univariate logistic regression analysis was performed to explore clinical factors for diagnosing pyonephrosis in the training cohort, and variables with *p* ≤ 0.10 in univariate analysis were considered candidates for multivariate logistic regression analysis to determine the independent predictors of pyonephrosis. Variables with *p* ≤ 0.05 in multivariate analysis were identified as independent clinical factors and used to establish the clinical model.

### 2.3. Image Acquisition and Segmentation

The non-contrast CT images were retrieved from the picture archiving and communication system for analysis. All patients underwent abdominal plain CT scans using 64-slice CT scanners (Discovery 750, GE Healthcare, Chicago, IL, USA; Aquilion ONE CT, Toshiba Medical Systems Corporation, Otawara, Tochigi, Japan) with the following parameters: tube voltage, 100–120 kV; automatic tube current modulation, 200–350 mA; rotation time, 0.5 s; matrix size, 512 × 512; scan slice thickness, 5 mm and reconstruction thickness, 1.25 mm.

The CT images, stored as DICOM files, were imported into the open-source software (ITK-SNAP, version 3.8.0, www.itksnap.org, accessed on 19 March 2024). The hydronephrosis or pyonephrosis regions of interest (ROIs) were manually delineated by a radiologist (reader 1) with 5 years of diagnostic abdominal imaging experience. Based on the ROI, the HU was measured and recorded. To ensure the reproducibility and stability of radiomics analysis [33], reader 1 carried out the identical procedure twice on 20 cases that were randomly selected after 2 weeks. A radiologist (reader 2) with 8 years of experience independently segmented 50 cases that were randomly selected. The intraclass correlation coefficient (ICC) was used to measure the intra-observer and inter-observer reliability. 

### 2.4. Feature Extraction, Selection, and Radiomics Model Building

To eliminate the influence of data from different sources on radiomics results, all the CT images underwent resampling (to a voxel size of 1 × 1 × 1 mm) and image normalization using a spline interpolation algorithm before extraction of radiomics features. A total of 1210 radiomic features were extracted from each ROI using the Pyradiomics soft-ware (version 3.0.1a1, https://pyradiomics.readthedocs.io/, accessed on 10 January 2024). The original images and two filtered images, namely laplacian of gaussian (LoG) and wavelet, were utilized for feature extraction. The feature categories included shape, first order, gray-level co-occurrence matrix (GLCM), gray-level dependence matrix (GLDM), gray-level run length matrix (GLRLM), gray-level size zone matrix (GLSZM), and neighborhood gray-tone difference matrix (NGTDM). 

**Figure 2:**
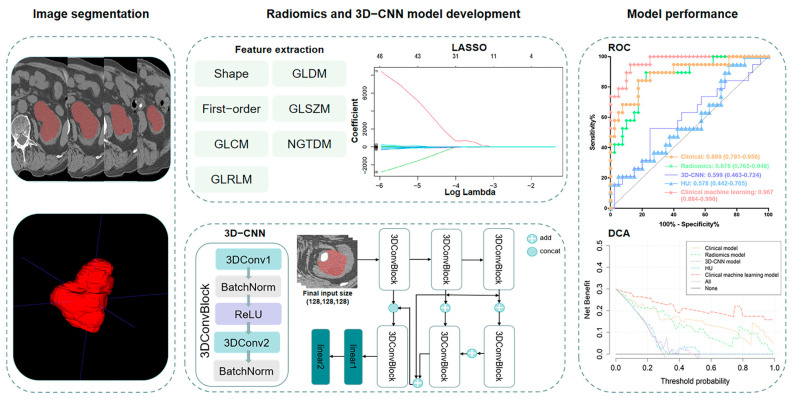
The development and performance evaluation of machine-learning models. The region of interest (ROI) was manually delineated. Radiomics features were extracted based on ROI, and features were selected through LASSO algorithm to calculate radiomics score. The coefficient distribution chart for radiomics features was displayed, where colored lines indicate different features and show the change in their coefficients across various levels of regularization. The 3D-CNN was also applied and the specific framework was listed. The performance of models was compared using ROC and DCA. GLDM, gray-level dependence matrix; GLSZM, gray-level size zone matrix; GLCM, gray-level co-occurrence matrix; NGTDM, neighborhood gray-tone difference matrix; GLRLM, gray-level run length matrix; LASSO, least absolute shrinkage and selection operator; 3D-CNN, three-dimensional convolutional neural network; ROC, receiver operating characteristic; DCA, decision curve analysis.

Three-step feature selection was employed to select the optimal radiomic features for diagnosing pyonephrosis. First, features with an ICC higher than 0.80 were selected. Second, the features were treated with the *t*-test between the pyonephrosis and hydronephrosis groups, and features with *p* ≤ 0.10 were retained for further analysis. Third, the least absolute shrinkage and selection operator (LASSO) method, which is suitable for the regression of high-dimensional data, was applied to further select features in the training cohort using 10-fold cross-validation. Finally, a radiomics score (Rad-score) was calculated for each patient using a linear combination of selected features weighted by their respective coefficients, and a radiomics model was then generated. The development of radiomics model is exhibited in Figure 2.

### 2.5. 3D-CNN Model Development

The 3D-CNN model was developed using Python (version 3.8, Python Software Foundation). Images were preprocessed on the whole cohort before inputting into the network. ROIs were extracted from images with the background pixel value set as 0. The windowing and image resize were adjusted using the bilinear interpolation for images and the nearest interpolation for masks. The final input size of images and masks into the CNN were all 128 × 128 × 128. The training and testing cohort is completely consistent with the radiomics analysis. To prevent overfitting, we also performed data augmentation including random flipping and rotation while training. The final 3D-CNN framework is shown in Figure 2.

### 2.6. Performance and Clinical Utility Assessment of Models

The discriminative performances of models were quantified by the receiver-operating characteristic (ROC) curve and the area under the curve (AUC) in both the training and testing cohorts. The Delong test was used to compare the AUC between the models. McNemar’s test was used to compare the sensitivity and specificity between the models. The clinical machine-learning model was established and evaluated with a combination of independent clinical factors and radiomics or CNN model with the best diagnostic performance. A nomogram was subsequently developed. To determine the clinical utility of the nomogram, decision curve analysis (DCA) was performed by calculating the net benefit at different threshold probabilities.

### 2.7. Statistical Analysis

Statistical analyses were performed using SPSS (version 25.0, IBM Corp, Chicago, IL, USA), MedCalc (version 15.8, Ostend, Belgium), and R software (version 3.6.3, R Foundation for Statistical Computing, Auckland, New Zealand). Continuous variables were presented as the median (interquartile range, IQR) and compared by the Mann–Whitney U test. Categorical variables were presented as numbers (percentages) and compared by the chi-squared analysis or Fisher’s exact test. All tests were two-sided and values of *p* ≤ 0.05 were considered statistically significant. 

## 3. Results

### 3.1. Patient Characteristics and Clinical Model Building

The characteristics of the patients in the training and testing cohorts are shown in Table 1. A total of 53 patients with pyonephrosis and 129 patients with hydronephrosis were enrolled and all patients were randomly assigned to the training cohort or the testing cohort in a ratio of 7:3 (123:59). There were no statistically significant differences in characteristics between the two cohorts (all *p* > 0.05). 

The results of univariate and multivariate logistic regression analysis for clinical factors associated with pyonephrosis in the training cohort are shown in Table 2. Multivariate analysis revealed that fever [odds ratio (OR): 24.436, 95% confidence interval (CI): 4.405–135.547, *p* < 0.001], blood neutrophils (OR: 1.107, 95% CI: 1.047–1.171, *p* < 0.001) and urine leukocytes (OR: 1.001, 95% CI: 1.000–1.002, *p* = 0.003) were independent clinical risk factors for pyonephrosis.

**Figure 3:**
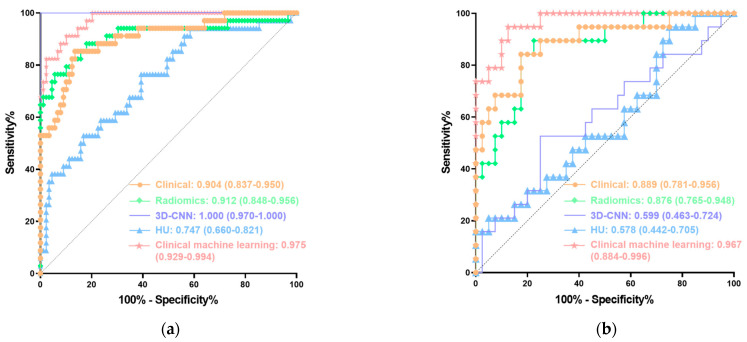
ROC curves of the clinical model, radiomics model, 3D-CNN model, HU, and clinical machine-learning model in the training (**a**) and testing (**b**) cohorts. 3D-CNN, three-dimensional convolutional neural network; ROC, receiver operating characteristic; AUC, area under the curve.

The clinical model based on the three clinical risk factors above exhibited an AUC of 0.904 (95% CI 0.837–0.950) with sensitivity and specificity of 0.853 and 0.865, respectively, in the training cohort (Table 3, Figure 3). In the testing cohort, it yielded an AUC of 0.889 (95% CI 0.781–0.956) with a sensitivity and specificity of 0.842 and 0.825, respectively, in the testing cohort (Table 4, Figure 3).

### 3.2. Construction of the Radiomics Model

A total of 1210 radiomics features were obtained from each ROI, of which 1111 (91.8%) radiomics features showed ICC > 0.80 in the inter-observer reproducibility analysis and 1161 (96.0%) radiomics features showed ICC > 0.80 in the intra-observer reproducibility analysis. After excluding the features with *p* > 0.10 in the t-test, the remaining 565 features were included in further analysis. By using the LASSO regression model, a total of eight features with non-zero coefficients were eventually selected as the input of the radiomics model. The Rad-score for each patient was calculated using the following formula:

Rad-score = (0.0243 × log-sigma-4-0-mm-3D_glszm_ZoneEntropy) + (0.3829 × log-sigma-5-0-mm 3D_glszm_ZoneEntropy) + (0.0053 × original_shape_MajorAxisLength) + (−6.0533 × original_shape_Sphericity) + 0.0093 × wavelet-HHL_glszm_GrayLevelNonUniformity) + (0.2480 × wavelet HLH_glszm_ZoneEntropy) + (3.0011 × wavelet-LLH_firstorder_Median) + (1.0610 × wavelet-LLH_glrlm_RunLengthNonUniformityNormalized) − 1.0104.

**Figure 4:**
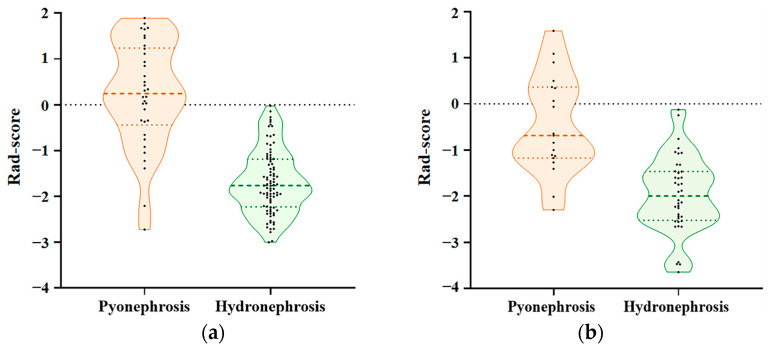
The Rad-score of the pyonephrosis and hydronephrosis groups in the training (**a**) and testing (**b**) cohorts. Rad-score: radiomics score.

There was a significant difference in the Rad-score between the pyonephrosis and hydronephrosis groups in both the training (0.22 vs. −1.69, *p* < 0.001) and testing (−0.46 vs. −1.96, *p* < 0.001) cohorts (Figure 4). The radiomics model showed an AUC of 0.912 (95% CI: 0.848–0.956) with sensitivity and specificity of 0.765 and 0.944, respectively, in the training cohort (Table 3, Figure 3). In the testing cohort, it achieved an AUC of 0.876 (95% CI: 0.765–0.948) with a sensitivity and specificity of 0.895 and 0.775, respectively (Table 4, Figure 3).

### 3.3. Development of the 3D-CNN Model

The 3D-CNN model was trained based on the training cohort data and showed an AUC of 1.000 (95% CI: 0.970–1.000) in the training cohort (Table 3, Figure 3). In the testing cohort, it achieved an AUC of 0.599 (95% CI: 0.463–0.724) with a sensitivity and specificity of 0.526 and 0.750, respectively (Table 4, Figure 3).

### 3.4. Comparison of Radiomics Model, 3D-CNN Model, and HU Performance

The HU was not significantly different between the training and testing cohorts (9.60 ± 7.54 vs. 11.04 ± 6.82, *p* = 0.214). In the training cohort, the HU was significantly different between the hydronephrosis and pyonephrosis groups (7.65 ± 6.31 vs. 14.68 ± 8.19, *p* < 0.001). However, the difference was not significant in the testing cohort (10.20 ± 6.45 vs. 12.81 ± 7.41, *p* = 0.173). Univariate and multivariate logistic regression analysis revealed that the HU was an independent risk factor for pyonephrosis in the training cohort [(OR with 95% CI): 1.148 (1.076–1.226), *p* < 0.001; 1.160 (1.057–1.273), *p* = 0.002]. The HU showed an AUC of 0.747 (95% CI: 0.660–0.821) with sensitivity and specificity of 0.765 and 0.607, respectively, in the training cohort (Table 3, Figure 3). In the testing cohort, it achieved an AUC of 0.578 (95% CI: 0.442–0.705) with a sensitivity and specificity of 0.947 and 0.250, respectively (Table 4, Figure 3).

The radiomics model (0.876 vs. 0.578, *p* = 0.002) and 3D-CNN model (0.599 vs. 0.578, *p* = 0.812) both exhibited a higher AUC than the measured HU in the testing cohort. The radiomics model outperformed the 3D-CNN model (AUC: 0.876 vs. 0.599; *p* = 0.003) (Table 4, Figure 3).

### 3.5. Establishment of the Clinical Machine-Learning Model

**Figure 5:**
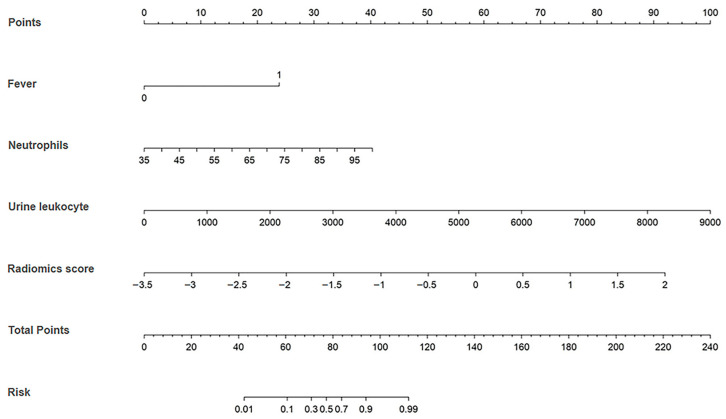
The developed nomogram based on the clinical machine-learning model to predict the risk of pyonephrosis. For example, locate the patient’s neutrophils count on the “Neutrophils” axis. Draw a line straight upward to the “Points” axis to determine how many points are received for the patient’s neutrophils indicators. Conduct a similar process for other indicators. Sum the points calculated for each of the factors and track down the added sum on the “Total Points” axis. Draw a line straight down to find the patient’s risk probability of pyonephrosis.

Radiomics model had the highest AUC in the testing cohort. Therefore, independent clinical risk factors, including fever, blood neutrophils, and urine leukocytes, were combined with the Rad-score by multivariate logistic regression to establish a final clinical machine-learning model. A nomogram was constructed based on this model. For each factor, we can obtain a point according to the patient’s clinical and radiomics information, and a higher total point reflects a corresponding case with a higher probability for the occurrence of pyonephrosis (Figure 5). 

The clinical machine-learning model showed an AUC of 0.975 (95% CI: 0.929–0.994) with sensitivity and specificity of 0.912 and 0.900, respectively in the training cohort. In the testing cohort, it exhibited an AUC of 0.967 (95% CI: 0.884–0.996) and sensitivity and specificity of 0.947 and 0.875, respectively. In addition, the clinical machine-learning model outperformed the clinical model and radiomics model in the training (AUC: 0.975 vs. 0.904, 0.912; *p* = 0.019, 0.065) and testing (AUC: 0.967 vs. 0.889, 0.876; *p* = 0.045, 0.061) cohorts, respectively (Table 3 and Table 4, Figure 3). 

**Figure 6:**
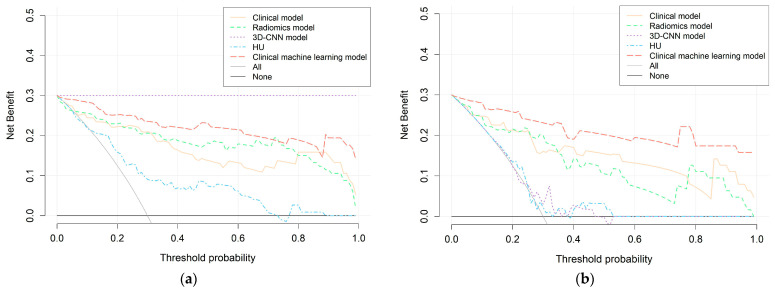
DCA curves of the clinical model, radiomics model, 3D-CNN model, HU, and clinical machine-learning model in the training (**a**) and testing (**b**) cohorts. The black line is the net benefit of assuming that all patients have pyonephrosis. The gray line is the net benefit of assuming that no patients have pyonephrosis. The red line, orange line, purple line, blue line, and green line represent the expected net benefit of predicting pyonephrosis using the clinical model, radiomics model, 3D-CNN model, HU, and clinical machine-learning model, respectively. DCA, decision curve analysis; 3D-CNN, three-dimensional convolutional neural network.

Furthermore, DCA indicated that in both the training and testing cohorts, the clinical machine-learning model provided a greater benefit than the clinical model and radiomic model in identifying calculous pyonephrosis across most of the threshold probabilities (Figure 6).

## 4. Discussion

In the present study, the radiomics model exhibited a good capability in identifying patients with calculous pyonephrosis, surpassing both the 3D-CNN model and HU in the testing cohort. The clinical machine-learning model, which integrates the Rad-score and three independent clinical factors, outperformed the individual clinical model. DCA further confirmed its clinical validity.

Regarding clinical indicators, the current work revealed that fever, blood neutrophils, and urine leukocytes were independent risk factors for pyonephrosis. Fever is considered an important indicative symptom of acute urinary tract infection [34] and previous research has similarly shown that the maximum body temperature is independently associated with the occurrence of pyonephrosis [12]. Neutrophils are the main cellular component of the host immune system. They were regarded as the primary mediator of innate immune defenses against invading microorganisms [35], which may explain why blood neutrophils were one of the independent risk factors for predicting pyonephrosis, as reported in the study of Wang et al. [36]. Two studies, by Wang et al. and Liu et al. [36,37], have indicated that an independent association was observed between urine leukocytes and pyonephrosis, consistent with our finding. Thus, when a patient presents with fever or elevated counts of blood neutrophils and urine leukocytes, the clinician should keep highly alert for the occurrence of pyonephrosis. Surprisingly, urine culture was not included in the establishment of the final model, likely because routine urine culture results cannot truly reflect the renal infection status due to the obstruction of the collecting system.

Several previous studies have reported that the HU can effectively differentiate pyonephrosis from hydronephrosis, with reported AUC values ranging from 0.780 to 0.854 [12,13,14]. However, our results indicated that the AUC of HU in the training and testing cohort were 0.747 and 0.578, respectively, which was lower than those previously reported. This may be attributed to the fact that previous studies measured the HU of pyonephrosis or hydronephrosis region in the single slice with the maximal collecting system surface area, whereas we measured the average HU across all slices to avoid subjective bias. Our results found that the radiomics and 3D-CNN models demonstrated higher AUC than the HU, indicating that radiomics and deep-learning features provide more valuable information. 

Although the 3D-CNN model can achieve classification between hydronephrosis and pyonephrosis, its AUC on the testing cohort is significantly inferior to the radiomics model. The relatively small sample size in this study might have hindered the effective training of the 3D-CNN model. Future improvements and validations of 3D-CNN models could benefit from larger, multicenter studies. In our study, the single radiomics model has already demonstrated a good ability to identify calculous pyonephrosis. The correlation between a single radiomic feature and biological information is difficult to comprehend and building multi-feature panels is a more common assessment method, as reported in several previous studies [38,39]. After radiomic analysis, the Rad-score was finally calculated based on selected eight radiomics features. Two morphology parameters were collected from original images, which may reveal that there are differences in the volume and shape between pyonephrosis and hydronephrosis. Two LoG-filtered radiomics features were included in the final radiomics model and many studies have reported that LoG features are closely related to lesion heterogeneity, microenvironment, and molecular biological information [16,40]. Purulent fluid includes bacteria, inflammatory cells, necrotic tissue, etc., and its composition is more complex than that of the fluid of hydronephrosis. In addition, the spatial colony-growth heterogeneity further increases the complexity of the pyonephrosis region [41]. The benefits of wavelet-filtered features in enhancing model competence were demonstrated by several prior reports [42,43,44]. The radiomics model includes four wavelet features and achieved an AUC of 0.912 and 0.876 in the training and testing cohort, respectively, demonstrating its good discrimination performance. In addition, of eight selected features, four features were GLSZM-based and several prior studies have reported that it could quantify image heterogeneity in terms of zones of contiguous voxels sharing the same grey level intensity [45]. All selected GLSZM features had positive coefficients, aligning with the notion that pyonephrosis fluid is more complex than that of hydronephrosis. 

In our research, the clinical machine-learning model achieved a higher AUC than the clinical model, which suggests that the clinical machine-learning model is more feasible for diagnosing pyonephrosis and may exert an indispensable effect on identifying the patients who have pyonephrosis but without certain suggestive clinical signs. In addition, comparisons and evaluations of each model by DCA further showed that the clinical machine-learning model resulted in net benefits of providing more than the clinical model or radiomics model across most of the threshold probabilities. A visualizable nomogram was generated based on the final model. With this easy-to-use scoring nomogram, clinicians could predict the individual risk probability of calculous pyonephrosis, which contributes to improving diagnostic efficiency and facilitating urgent drainage and anti-infection treatment to prevent patients from disastrous outcomes.

This study has several limitations. First, the study was retrospective in design and potential selection bias was inevitable. Hence, prospective studies are needed. Second, this investigation was conducted in a single institution and no independent external dataset was available for validation, the clinical application and generalization of the nomogram still need to be further improved and validated by multi-center studies with larger sample sizes. Third, we will attempt to apply deep-learning algorithms to achieve automatic segmentation of hydronephrosis or pyonephrosis regions to simplify clinical operations. Fourth, dynamic analysis based on machine learning has already been widely applied in single-particle tracking techniques and has garnered significant attention in recent years [46]. Our study mainly focused on the analysis of static images, but a potential future research direction is to incorporate time-series data to analyze the dynamic evolution of abnormalities, which contributes to the early diagnosis of pyonephrosis.

## 5. Conclusions

In summary, the radiomics and 3D-CNN models showed better performance than the HU, which suggests that the high-throughput features may offer more valuable internal information about lesions. In addition, the radiomics model was more effective in differentiating calculous pyonephrosis from uninfected hydronephrosis than the 3D-CNN model. The clinical machine-learning model constructed by combining Rad-score and clinical risk factors outperformed the individual clinical model, which provides a non-invasive and comprehensive diagnostic method for pyonephrosis. It can also be helpful for clinicians to identify patients who have pyonephrosis but without certain suggestive clinical signs and implement urgent or more appropriate treatment to prevent patients from having a poor prognosis. In the future, this clinical machine-learning model needs to be validated by multi-center large sample studies. In addition, applying advanced artificial intelligence architecture to achieve one-stop analysis from acquiring CT images to diagnosing calculous pyonephrosis, will be more conducive to assisting clinical decision-making.

## Figures and Tables

**Table 1:** Characteristics of the patients in the training and testing cohorts.

Characteristics	Training Cohort (*n* = 123)	Testing Cohort (*n* = 59)	*p* Value
Age (years), median [IQR]	54 (47–59)	50 (42–60)	0.138
Gender, n (%)			0.479
Male	64 (52.0%)	34 (57.6%)	
Female	59 (48.0%)	35 (42.4%)	
BMI (kg∙m^−2^)	23.5 (21.5–25.5)	23.1 (21.6–26.0)	0.673
Fever			0.689
Yes	15 (12.2%)	6 (10.2%)	
No	108 (87.8%)	53 (89.8%)	
Renal colic			0.243
Yes	68 (55.3%)	38 (64.4%)	
No	55 (44.7%)	21 (35.6%)	
Hypertension			0.110
Yes	39 (31.7%)	12 (20.3%)	
No	84 (68.3%)	47 (79.7%)	
Diabetes			0.435
Yes	20 (16.3%)	7 (11.9%)	
No	103 (83.7%)	52 (88.1%)	
History of stone surgery			0.888
Yes	43 (35.0%)	20 (33.9%)	
No	80 (65.0%)	39 (66.1%)	
Stone laterality			0.710
Left	62 (50.4%)	28 (47.5%)	
Right	61 (49.6%)	31 (52.5%)	
Stone location			0.423
Renal calculus	30 (24.4%)	19 (32.2%)	
Ureteral calculus	31 (25.2%)	14 (23.7%)	
Renal and ureteral calculus	62 (50.4%)	26 (44.1%)	
Stone size (mm)	15.2 (11.1–21.5)	16.0 (11.0–19.0)	0.807
Levels			0.486
Mild/Moderate	90 (73.2%)	46 (78.0%)	
Severe	33 (26.8%)	13 (22.0%)	
WBC (×10^9^/L)	6.06 (5.05–8.09)	6.42 (5.24–8.11)	0.544
Neutrophils (×10^9^/L)	58.9 (53.5–68.2)	60.1 (53.6–68.4)	0.709
Platelet (×10^9^/L)	226 (182–276)	232 (171–279)	0.569
Urine leukocyte (/µL)	88.6 (37.8–321.2)	150.4 (41.1–1111.9)	0.129
Urinary nitrite			0.507
Positive	12 (9.8%)	4 (6.8%)	
Negative	111 (90.2%)	55 (93.3%)	
Urine culture			0.693
Positive	26 (21.1%)	14 (23.7%)	
Negative	97 (78.9%)	45 (76.3%)	

Abbreviations: IQR: interquartile range; BMI: body mass index; WBC: white blood cell.

**Table 2:** Univariate and multivariate logistic regression analysis of clinical factors for pyonephrosis.

Characteristics	Univariate Analysis		Multivariate Analysis	
	OR (95% CI)	*p* Value	OR (95% CI)	*p* Value
Age (years)	1.011 (0.978–1.044)	0.524		
Gender	2.168 (0.965–4.870)	0.061		
BMI (kg∙m^−2^)	0.924 (0.822–1.039)	0.188		
Fever	15.636 (4.058–60.258)	<0.001 *	24.436 (4.405–135.547)	<0.001 *
Renal colic	1.444 (0.644–3.236)	0.373		
Hypertension	1.500 (0.655–3.436)	0.338		
Diabetes	1.974 (0.727–5.361)	0.182		
History of stone surgery	1.720 (0.764–3.872)	0.190		
Stone laterality	1.203 (0.545–2.656)	0.646		
Stone location	0.705 (0.319–1.562)	0.390		
Stone size (mm)	1.038 (0.988–1.092)	0.140		
Hydronephrosis	3.114 (1.328–7.300)	0.009 *		
WBC (×10^9^/L)	1.247 (1.092–1.425)	0.001 *		
Neutrophils (×10^9^/L)	1.107 (1.061–1.155)	<0.001 *	1.107 (1.047–1.171)	<0.001 *
Platelet (×10^9^/L)	1.005 (1.001–1.009)	0.033 *		
Urine leukocyte (/µL)	1.001 (1.000–1.002)	0.002 *	1.001 (1.000–1.002)	0.003 *
Urinary nitrite	2.020 (0.594–6.864)	0.260		
Urine culture	2.922 (1.181–7.228)	0.020 *		

Abbreviations: OR: odds ratio; CI: confidence interval; BMI: body mass index; WBC: white blood cell. The character of “*” indicates a statistically significant difference.

**Table 3:** Diagnostic performances of different models in the training cohort.

Model	Training Cohort (*n* = 123)					
	AUC (95% CI)	*p^α^* Value	Sensitivity	*p^β^* Value	Specificity	*p^γ^* Value
Clinical model	0.904 (0.837–0.950)	0.866 ^a^	0.853	0.549 ^a^	0.865	0.210 ^a^
Radiomics model	0.912 (0.848–0.956)	0.065 ^b^	0.765	0.063 ^b^	0.944	0.375 ^b^
3D-CNN model	1.000 (0.970–1.000)	0.018 ^c^*	1.000	/	1.000	/
HU	0.747 (0.660–0.821)	0.007 ^d^*	0.765	>0.999 ^d^	0.607	<0.001 ^d^*
Clinical machine-learning model	0.975 (0.929–0.994)	0.019 ^e^*	0.912	0.687 ^e^	0.900	0.607 ^e^

Abbreviations: AUC: area under the curve; CI: confidence interval; 3D-CNN: three-dimensional convolutional neural network; HU: Hounsfield Unit. *p****^α^***: the comparison of AUC; *p^β^*: the comparison of sensitivity; *p^γ^****:*** the comparison of specificity. a: the comparison between clinical model and radiomics model; b: the comparison between radiomics model and clinical machine-learning model; c: the comparison between radiomics model and 3D-CNN model; d: the comparison between radiomics model and HU; e: the comparison between clinical model and clinical machine-learning model. The 3D-CNN model is based on training cohort to learn image features of hydronephrosis or pyonephrosis and is not suitable for comparing sensitivity and specificity with other models using McNemar’s test. The character of “*” indicates a statistically significant difference.

**Table 4:** Diagnostic performances of different models in the testing cohort.

Model	Testing Cohort (*n* = 59)					
	AUC (95% CI)	*p^α^* Value	Sensitivity	*p^β^* Value	Specificity	*p^γ^* Value
Clinical model	0.889 (0.781–0.956)	0.852 ^a^	0.842	>0.999 ^a^	0.825	0.774 ^a^
Radiomics model	0.876 (0.765–0.948)	0.061 ^b^	0.895	>0.999 ^b^	0.775	0.219 ^b^
3D-CNN model	0.599 (0.463–0.724)	0.003 ^c^*	0.526	0.039 ^c^*	0.750	>0.999 ^c^
HU	0.578 (0.442–0.705)	0.002 ^d^*	0.947	>0.999 ^d^	0.250	<0.001 ^d^*
Clinical machine-learning model	0.967 (0.884–0.996)	0.045 ^e^*	0.947	0.500 ^e^	0.875	0.687 ^e^

Abbreviations: AUC: area under the curve; CI: confidence interval; 3D-CNN: three-dimensional convolutional neural network; HU: Hounsfield Unit. *p^α^*: the comparison of AUC; *p^β^*: the comparison of sensitivity; *p^γ^*: the comparison of specificity. a: the comparison between clinical model and radiomics model; b: the comparison between radiomics model and clinical machine-learning model; c: the comparison between radiomics model and 3D-CNN model; d: the comparison between radiomics model and HU; e: the comparison between clinical model and clinical machine-learning model. The character of “*” indicates a statistically significant difference.

## Data Availability

All data are available in this article.
